# Impact of different paraspinal muscle mass on the prognosis of ACDF—a finite element analysis

**DOI:** 10.3389/fbioe.2026.1743924

**Published:** 2026-02-23

**Authors:** Haojun Cui, Mengmeng Zhou, Yi Gong, Hongjie Zhang, Tengfei Zhang, Xin Tan, Xusheng Bi, Maosen Zhang, Xuan Wang, Zehua Jiang, Rusen Zhu

**Affiliations:** 1 Department of Spine Surgery, Tianjin Union Medical Center, The First Affiliated Hospital of Nankai University, Tianjin, China; 2 Department of Spine Surgery, Tianjin Union Medical Center, Tianjin Medical University, Tianjin, China; 3 Department of Orthopedics, The People’s Hospital of Dehong/Kunming Medical University Affiliated Dehong Hospital, Mangshi, China

**Keywords:** ACDF, cervical spondylotic myelopathy, finite element analysis, paraspinal muscles, postoperative cervical spine

## Abstract

**Introduction:**

Cervical spondylotic myelopathy (CSM) is a common degenerative disease of the cervical spine, for which anterior cervical discectomy and fusion (ACDF) serves as an effective surgical treatment. Recent studies have suggested that the quality of the paraspinal muscles, particularly the multifidus muscle, is closely related to postoperative outcomes; However, biomechanical evidence remains limited. The aim of this study is to investigate the biomechanical impact of varying paraspinal muscle mass on the cervical spine following ACDF.

**Methods:**

A finite element model of the cervical spine, including vertebrae, intervertebral discs, ligaments, and implants (cage and screws), was developed based on CT data from a healthy volunteer. Three models simulating different postoperative states of the multifidus muscle were constructed: a postoperative muscle training model (120% muscle quality), a postoperative muscle atrophy model (80% muscle quality), and a control model (100% muscle quality). Flexion, extension, lateral bending, and rotational loads were applied to each model to analyze changes in adjacent segment disc pressure, implant stress distribution, capsular ligament stress, and range of motion (ROM).

**Results:**

In the finite element models of different muscle quality groups after ACDF, the muscle atrophy model (80% muscle quality) showed a general increase in the intervertebral disc pressure of adjacent segments, especially during flexion-extension movements, which indicates an elevated risk of degeneration. Meanwhile, the stress values of implants such as cages and screws were increased, with more significant elevation observed during flexion and rotation. The capsular ligament stress was also elevated in the muscle atrophy model, and load overload was prone to occur during extension and rotation. In addition, muscle atrophy could lead to an increase in the ROM of adjacent segments. In contrast, all biomechanical indices of the muscle exercise model (120% muscle quality) were superior to those of the normal model.

**Conclusion:**

Paraspinal muscle quality is a critical factor influencing biomechanical stability after ACDF. Muscle atrophy may increase the risk of adjacent segment degeneration and implant failure, while muscle strengthening contributes to enhancing postoperative stability. These results support that preoperative evaluation of paraspinal muscle status and targeted postoperative muscle strength training hold significant clinical implications for improving surgical prognosis.

## 1 Introduction

Cervical spondylotic myelopathy (CSM) refers to degenerative changes in the cervical intervertebral discs and subsequent pathological effects on the spinal cord, nerve roots, vertebral arteries, sympathetic nerves, and other surrounding tissues (Davies et al., 2018; Peng and DePalma, 2018). Natural history studies indicate that the majority of patients experience neurological deterioration over time, with up to 56% of individuals reporting difficulties in performing daily living activities within 10 years of initial diagnosis if CSM is not treated surgically (Abdelmalek et al., 2025). Both conservative and surgical interventions are primary treatment options for cervical spondylosis.

Over the past decades, anterior cervical discectomy and fusion (ACDF) has been proven effective in improving clinical outcomes for degenerative cervical conditions, such as cervical radiculopathy and cervical spondylotic myelopathy (Chang et al., 2022; Louie et al., 2022).

The multifidus muscle (MF), a major component of the cervical paraspinal muscles (PSM), serves as a posterior tension band that provides structural support to the cervical spine and discs, participates in transmitting axial compressive forces, and plays a vital role in cervical motion and stability (Fernández-de-las-Peñas et al., 2008; Cheng et al., 2011). It helps maintain normal physiological curvature and mechanical balance while reducing abnormal intervertebral stress. The condition of the multifidus muscle has been shown to correlate positively with postoperative outcomes in ACDF (Wong et al., 2021; Caffard et al., 2024; Peterson et al., 2019). Therefore, this study initially explored the MF as a representative of the cervical PSM. However, these clinical correlations lack direct biomechanical support, and conventional biomechanical testing methods are inadequate for dynamically simulating the impact of varying muscle conditions on surgical prognosis (Tamai et al., 2019).

Finite element analysis (FEA), as an efficient simulation technique, is widely used in medical applications, particularly in evaluating cervical implants (Xu et al., 2025). Its advantages include non-invasive physiological stress analysis, accurate simulation of complex anatomical structures and mechanical loads (Grohganz et al., 2024), significant reduction in medical device testing cost and time (Stott and Driscoll, 2023), decreased reliance on physical prototyping and bench testing, and avoidance of ethical issues associated with *in vivo* and *in vitro* experiments (Dreischarf et al., 2014). By constructing a three-dimensional cervical model, this study employs FEA to quantitatively analyze key postoperative indicators such as disc stress, implant loading, and capsular strain, thereby providing a scientific basis for assessing long-term implant stability and optimizing clinical strategies.

Therefore, this study aims to use FEA to evaluate the biomechanical stability of the cervical spine–implant system under varying muscle mass conditions, using a normal muscle model as a control, to provide more accurate guidance for clinical practice.

## 2 Materials and methods

### 2.1 Construction of a finite element model of C2–7 in healthy adults

The studies involving human participants were reviewed and approved by Tianjin Union Medical Center, Tianjin, China (No. 2025-C47). This study utilized data from a healthy male volunteer, and a finite element model of the cervical spine was constructed following the modeling methodology reported by Chen et al. (2024). Strict quality control of radiological data was performed: all images were thoroughly reviewed by an experienced physician to exclude conditions such as cervical spondylosis, fractures, spinal deformities, osteoporosis, tumors, and other disorders that may affect spinal biomechanical properties, thereby ensuring data validity (Liu et al., 2024; Xu et al., 2022; Hou et al., 2023).

A 64-slice spiral CT scanner was used to acquire transverse sectional images of the volunteer’s cervical spine, which were stored in DICOM format. The DICOM files were imported into Mimics 21.0 to reconstruct three-dimensional vertebral models. Simultaneously, intervertebral discs and nucleus pulposus structures were constructed using 3-Matic 12.0. The preliminary model was then exported to Geomagic Studio 2012 for optimization: initial adjustments and surface smoothing were performed by removing redundant spikes, relaxing the model structure, and reducing image noise. Further refinement was achieved through steps such as defining contour threads and constructing surface patches.

The optimized model was subsequently imported into Hypermesh for the construction and meshing of key components, including the joint capsules, endplates, and annulus fibrosus, with a mesh size set to 1 mm. The complete finite element model was finally imported into Ansys 2024 for subsequent strain and stress analyses. Material parameters used in the model are listed in Table 1, with reference to studies by Sun et al. (2022), Wang et al. (2016), Purushothaman et al. (2021), and Schmidt et al. (2007). In terms of material property definitions, cortical bone, cartilage endplates, cancellous bone, and articular cartilage were modeled with tetrahedral elements and isotropic elastic properties. The nucleus pulposus and annulus fibrosus ground substance adopted tetrahedral elements with Mooney–Rivlin hyperelastic behavior. Annulus fibrosus fibers were defined as orthotropic nonlinear elastic materials integrated into the disc. Ligaments were assigned nonlinear curve characteristics (tension-only response) with appropriate elements. Implants (cage and screws) used tetrahedral elements with isotropic elastic properties. The multifidus muscle was represented by hexahedral elements as a Neo-Hookean nonlinear hyperelastic and incompressible material.

**TABLE 1:** Material properties of cervical tissue.

Component	Element type	Material type	Material parameters
Cortical bone	Tetrahedral element	Isotropic elasticity	E = 12 GPa V = 0.29
Cancellous bone	Tetrahedral element	Isotropic elasticity	E = 100 MPa V = 0.29
Articular cartilage	Tetrahedral element	Isotropic elasticity	E = 5 MPa V = 0.3
Cartilage endplate	Tetrahedral element	Isotropic elasticity	E = 5.4 GPa V = 0.3
Nucleus	Tetrahedral element	Mooney-Rivlin	C10 = 0.12, C01 = 0.03
Annulus ground	Tetrahedral element	Mooney-Rivlin	C10 = 0.18, C01 = 0.045
Cage	Tetrahedral element	Isotropic elasticity	E = 3 GPa V = 0.4
Screws	Tetrahedral element	Isotropic elasticity	E = 96 GPa V = 0.36
Multifidus muscles	Hexahedral element	Neo-Hookean	Mu = 0.032 MPa D1 = 18

### 2.2 Mesh convergence test

To verify the independence of the calculation results from mesh size, we performed mesh sensitivity analysis on the muscle model (100%), comparing the changes in intradiscal pressure of the C4-C5 segment and cage stress under mesh sizes of 1.0 mm, 1.5 mm, and 2.0 mm. The results showed that when the mesh size was refined to below 1.5 mm, the changes in the results were less than 5%, indicating that the mesh size of 1.0 mm is sufficient to meet the requirements of calculation accuracy.

### 2.3 Construction of the cervical spine-implant system model under different muscle mass

**FIGURE 1:**
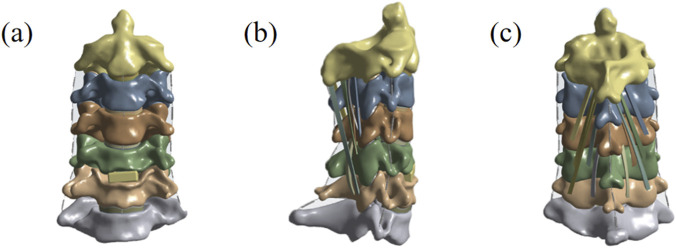
**(a)** Anteroposterior view following cervical ACDF surgery. **(b)** Lateral view following cervical ACDF surgery. **(c)** Posterior view following cervical ACDF surgery.

Based on the aforementioned normal cervical spine model, a postoperative finite element model was further developed: the actual surgical procedure was simulated by selectively removing the C5/6 intervertebral disc and the corresponding anterior longitudinal ligament. It was assumed that the bone graft had achieved complete fusion with the adjacent vertebrae, ultimately resulting in the construction of a finite element model following ACDF. A schematic diagram of the specific model is shown in Figure 1.

**FIGURE 2:**
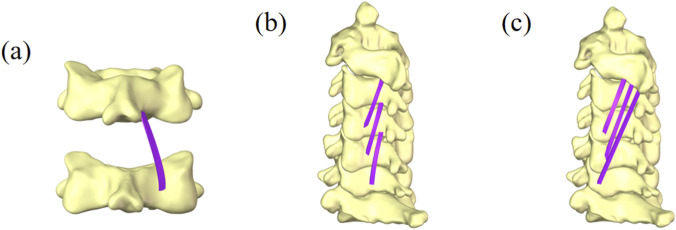
Finite element model of the cervical spine with the multifidus muscle. **(a)** View of part of the deep multifidus muscle. **(b)** Lateral view of the deep multifidus muscle. **(c)** Lateral view of the superficial multifidus muscle.

To evaluate the impact of morphological changes in the MF on cervical spine tissues, a finite element model of the cervical spine incorporating the MF was constructed (Anderson et al., 2005), as shown in Figure 2.

**FIGURE 3:**
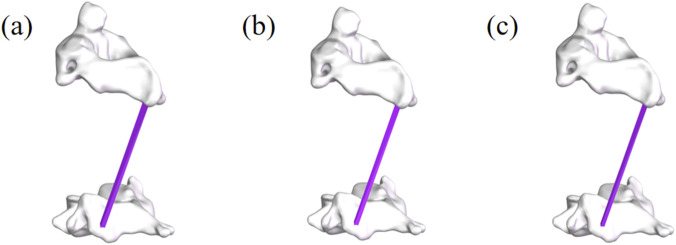
Partial view of multifidus muscle atrophy. **(a)** Finite element model with 120% content of multifidus muscle. **(b)** Finite element model with 100% content of multifidus muscle. **(c)** Finite element model with 80% content of multifidus muscle.

Referring to previous literature (Kang et al., 2021), finite element models with MF content of 120%, 100%, and 80% were established as shown in Figure 3, representing the postoperative muscle exercise (120%), the normal muscle condition (100%), and postoperative muscle atrophy (80%). Among them, the models with 80% and 120% MF content were generated by uniformly scaling the cross-sectional area of the multifidus muscle in the base model (100%), with scaling factors of 0.8 and 1.2, respectively. The scaling preserved the original shape and attachment points of the muscle. The total CSA of the bilateral multifidus muscles at the C5-C6 level in the 100% baseline model was 2098.53 mm^2^. Based on this, the CSAs of the 80% and 120% models were 1678.82 mm^2^ and 2518.24 mm^2^, respectively. In addition, the multifidus muscle was defined as a nonlinear hyperelastic and incompressible material (Dao et al., 2014). The contact between the multifidus muscle and bone was set as a tied interface.

### 2.4 Loading conditions and validation of the finite element model

In the cervical spine finite element models, a concentrated vertically downward force of 50 N was applied on the upper surface of the C1 vertebra to simulate the head weight. Meanwhile, a pure moment of 1 Nm was exerted at the reference point on the upper surface of the C1 vertebra to simulate four physiological motion conditions: flexion, extension, lateral bending, and rotation. Additionally, the C7 vertebra and the lower ends of the multifidus muscles were subjected to fully fixed constraints to simulate the fixed support conditions of the lower cervical spine under physiological states.

Due to ethical and technical challenges associated with the direct *in vivo* measurement of intradiscal pressure and implant stress, this study primarily adopted a widely accepted indirect validation method, namely, verifying the ROM, to ensure the reliability of the overall biomechanical behavior of the model. The loading protocol followed the approach established in Sun et al.’s study (Sun et al., 2023): an equivalent head weight load was applied to the model, along with a 1 Nm moment, to simulate and record the mechanical responses of the cervical spine under four physiological motion conditions—flexion, extension, lateral bending, and rotation. The results were compared with those reported in previous literature to validate the model’s effectiveness.

## 3 Results

### 3.1 Validation of the complete cervical spine finite element model

**FIGURE 4:**
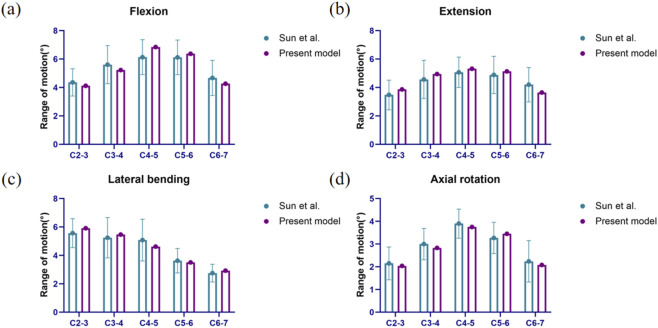
Comparison of ROM. **(a)** Results of the ROM under flexion. **(b)** Results of the ROM under extension. **(c)** Results of the ROM under lateral bending. **(d)** Results of the ROM under Axial rotation.

The ROM values in most segments of our model fell within one standard deviation of the mean values reported in previous *in vitro* studies (as shown in Figure 4). Furthermore, the trends across all segments remained consistent, confirming that our finite element model accurately represents the kinematic characteristics of the cervical spine and is suitable for analyzing the biomechanical effects of subsequent ACDF procedures (Sun et al., 2023).

### 3.2 Intradiscal pressure changes at adjacent segments

**FIGURE 5:**
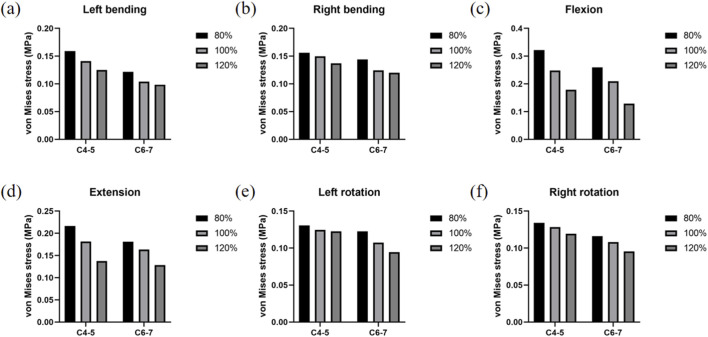
Intervertebral disc stress result. **(a)** Strain changes of the intervertebral disc under left bending. **(b)** Strain changes of the intervertebral disc under right bending. **(c)** Strain changes of the intervertebral disc under flexion. **(d)** Strain changes of the intervertebral disc under extension. **(e)** Strain changes of the intervertebral disc under left rotation. **(f)** Strain changes of the intervertebral disc under right rotation.

**FIGURE 6:**
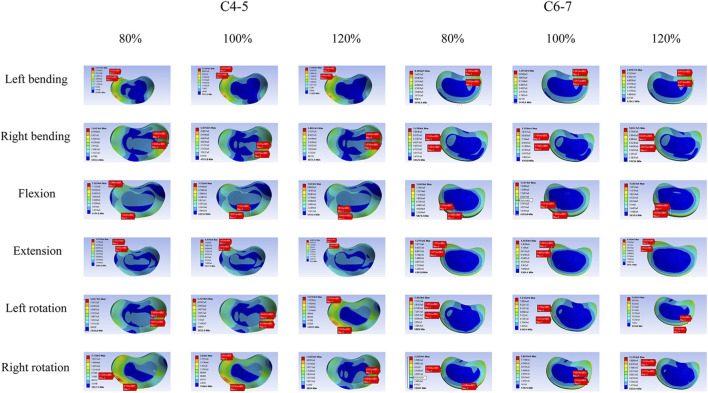
The stress nephogram of the adjacent discs. (L) C4-5; (R) C6-7.

Figure 5 shows the values of intradiscal pressure changes at the adjacent segment under different working conditions, while Figure 6 displays the disc pressure values at the adjacent segments across the three finite element models under all loading conditions. The results indicate significant differences among the models. Specifically, the muscle atrophy model exhibited increased intradiscal pressure, suggesting that higher muscle content corresponds to lower disc pressure—particularly under flexion and extension loads. This implies a reduction in the compensatory capacity of the paraspinal muscles. In contrast, the muscle exercise model demonstrated a more favorable pressure distribution, with values lower than those in the control muscle model. The detailed intradiscal pressure changes relative to the control model are as follows:

In the 80% muscle quality model, C4-5 and C6-7 Intradiscal pressures were consistently elevated across all motions:

Left flexion: +12.87% (C4-5), +17.08% (C6-7)

Right flexion: +4.07% (C4-5), +15.80% (C6-7)

Flexion: +29.59% (C4-5), +23.65% (C6-7)

Extension: +19.33% (C4-5), +10.76% (C6-7)

Left rotation: +4.85% (C4-5), +14.12% (C6-7)

Right rotation: +4.56% (C4-5), +7.42% (C6-7)

In contrast, the 120% muscle quality model exhibited reduced C4-5 and C6-7 disc pressures in all motions:

Left flexion: −11.23% (C4-5), −5.36% (C6-7)

Right flexion: −8.59% (C4-5), −3.55% (C6-7)

Flexion: −28.04% (C4-5), −38.69% (C6-7)

Extension: −24.23% (C4-5), −21.49% (C6-7)

Left rotation: −1.70% (C4-5), −11.86% (C6-7)

Right rotation: −7.01% (C4-5), −11.57% (C6-7)

### 3.3 Stress changes in the cage and screws

**FIGURE 7:**
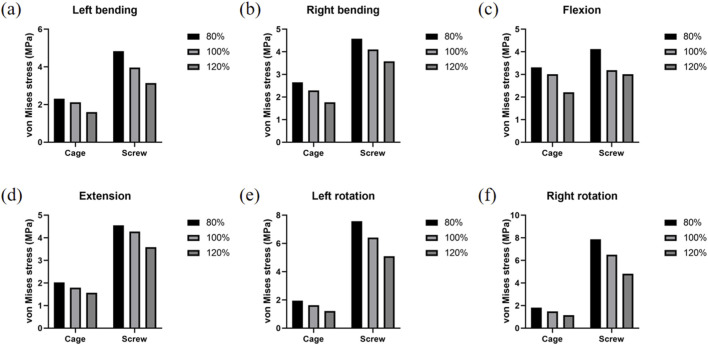
Stress changes in cage and screw. **(a)** Strain changes of the cage and screw under left bending. **(b)** Strain changes of the cage and screw under right bending. **(c)** Strain changes of the cage and screw under flexion. **(d)** Strain changes of the cage and screw under extension. **(e)** Strain changes of the cage and screw under left rotation. **(f)** Strain changes of the cage and screw under right rotation.

As shown in Figure 7, the stress distribution within the cage and pedicle screws was highly dependent on muscle condition and load direction. Compared to the control group, the muscle atrophy model (80%) exhibited higher stress values in both the cage and screws, with particularly noticeable stress concentration during flexion and rotation. The detailed stress changes relative to the control model are as follows:

In the 80% muscle quality model, cage and screw stresses were consistently elevated across all motions:

Left flexion: +8.96% (Cage), +21.91% (Screw)

Right flexion: +15.93% (Cage), +11.67% (Screw)

Flexion: +10.11% (Cage), +29.48% (Screw)

Extension: +12.88% (Cage), +6.50% (Screw)

Left rotation: +19.52% (Cage), +17.92% (Screw)

Right rotation: +22.10% (Cage), +21.08% (Screw)

In contrast, the 120% muscle quality model exhibited reduced cage and screw stresses in all motions:

Left flexion: −24.48% (Cage), −20.65% (Screw)

Right flexion: −22.74% (Cage), −12.65% (Screw)

Flexion: −26.76% (Cage), −5.40% (Screw)

Extension: −12.52% (Cage), −16.00% (Screw)

Left rotation: −25.84% (Cage), −20.67% (Screw)

Right rotation: −22.58% (Cage), −25.92% (Screw)

This indicates an increased risk of mechanical failure or loosening of the implants. In contrast, the muscle exercise model (120%) showed generally reduced stress levels and lower peak stress values across all loading scenarios.

### 3.4 Stress changes in the joint capsule

**FIGURE 8:**
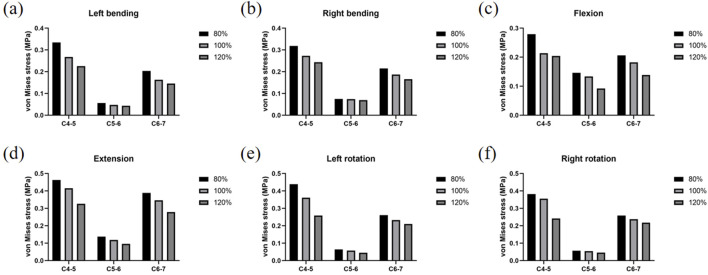
Strain changes of joint capsules. **(a)** Strain changes of joint capsules under left bending. **(b)** Strain changes of joint capsules under right bending. **(c)** Strain changes of joint capsules under flexion. **(d)** Strain changes of joint capsules under extension. **(e)** Strain changes of joint capsules under left rotation. **(f)** Strain changes of joint capsules under right rotation.

As shown in Figure 8, the stress experienced by the joint capsule varied significantly depending on muscle condition and load type. The muscle atrophy model (80%) consistently exhibited higher joint capsule stress, particularly under extension and rotation conditions, indicating a potential risk of overload. The detailed stress changes relative to the control model are as follows:

In the 80% muscle quality model, C4-5, C5-6, and C6-7 joint capsule stresses were consistently elevated across all motions:

Left flexion: +24.89% (C4-5), +18.08% (C5-6), +25.16% (C6-7)

Right flexion: +16.62% (C4-5), +0.99% (C5-6), +15.22% (C6-7)

Flexion: +30.78% (C4-5), +9.52% (C5-6), +12.99% (C6-7)

Extension: +11.44% (C4-5), +16.02% (C5-6), +12.37% (C6-7)

Left rotation: +21.37% (C4-5), +12.05% (C5-6), +12.24% (C6-7)

Right rotation: +7.32% (C4-5), +5.64% (C5-6), +8.61% (C6-7)

In contrast, the 120% muscle quality model exhibited reduced C4-5, C5-6, and C6-7 joint capsule stresses in all motions:

Left flexion: −15.58% (C4-5), −9.37% (C5-6), −10.42% (C6-7)

Right flexion: −10.81% (C4-5), −6.05% (C5-6), −11.23% (C6-7)

Flexion: −4.34% (C4-5), −31.18% (C5-6), −24.05% (C6-7)

Extension: −21.49% (C4-5), −18.55% (C5-6), −19.53% (C6-7)

Left rotation: −28.36% (C4-5), −21.09% (C5-6), −9.41% (C6-7)

Right rotation: −32.00% (C4-5), −14.82% (C5-6), −8.59% (C6-7)

### 3.5 ROM of the cervical spine

**FIGURE 9:**
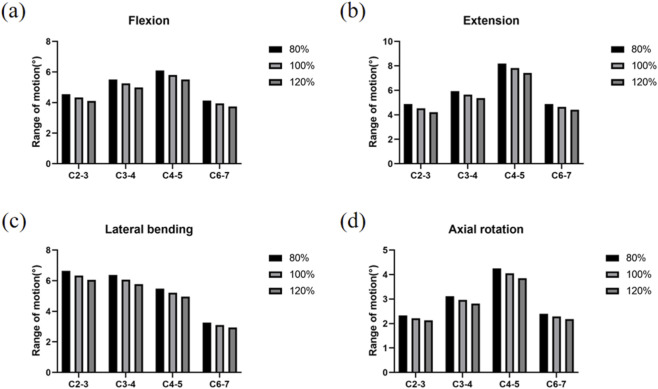
Comparison of ROM. **(a)** Results of the ROM under flexion. **(b)** Results of the ROM under extension. **(c)** Results of the ROM under lateral bending. **(d)** Results of the ROM under axial rotation.

The results of changes in the ROM of the cervical spine are shown in Figure 9. The content of paraspinal muscles had a significant impact on the ROM of the adjacent segments. Under the four physiological loading conditions—flexion, extension, lateral bending, and rotation—the muscle atrophy model (80%) exhibited a significant increase in ROM at the C5–C6 segment compared to the control muscle model (100%), suggesting potential hypermobility at the adjacent segments. In contrast, the muscle exercise model (120%) demonstrated lower ROM values under the same loading conditions than the control group, indicating improved segmental stability. The detailed ROM changes relative to the control model are as follows:

In the 80% muscle quality model, C2-3, C3-4, C4-5, and C6-7 ROM were consistently elevated across all motions:

Flexion: +7.95% (C2-3), +4.96% (C3-4), +4.99% (C4-5), +4.95% (C6-7)

Extension: +4.85% (C2-3), +4.76% (C3-4), +4.82% (C4-5), +4.82% (C6-7)

Lateral bending: +5.06% (C2-3), +4.94% (C3-4), +4.98% (C4-5), +5.14% (C6-7)

Rotation: +4.50% (C2-3), +5.39% (C3-4), +5.19% (C4-5), +9.17% (C6-7)

In contrast, the 120% muscle quality model exhibited reduced C2-3, C3-4, C4-5, and C6-7 ROM in all motions:

Flexion: −6.84% (C2-3), −5.13% (C3-4), −4.87% (C4-5), −5.16% (C6-7)

Extension: −5.08% (C2-3), −5.14% (C3-4), −4.99% (C4-5), −5.08% (C6-7)

Lateral bending: −4.42% (C2-3), −5.11% (C3-4), −5.36% (C4-5), −5.47% (C6-7)

Rotation: −4.05% (C2-3), −5.05% (C3-4), −4.94% (C4-5), −4.80% (C6-7)

## 4 Discussion

CSM represents a significant public health challenge. Treatment strategies are broadly categorized into conservative management and surgical intervention (Burneikiene et al., 2015). ACDF aims to effectively alleviate neural compression, restore intervertebral disc height, correct sagittal alignment, and ultimately improve patients’ clinical symptoms (Sun et al., 2024).

In recent years, numerous studies have indicated that the preoperative quality of cervical paraspinal muscles is closely associated with the postoperative prognosis of ACDF (Caffard et al., 2024; Harinathan et al., 2024). Fernández et al. demonstrated that the fat infiltration rate of the multifidus muscle is significantly correlated with poor patient prognosis (Fernández-de-las-Peñas et al., 2008); meanwhile, findings from Caffard et al. emphasized that paraspinal muscle quality and sagittal parameters have a certain correlation with complications following ACDF in patients (Caffard et al., 2024). Interestingly, our study also revealed a correlation between paraspinal muscle quality and sagittal parameters such as the Cobb angle (Cheng et al., 2025), suggesting that paraspinal muscle quality is a critical factor influencing the prognosis of ACDF. However, these studies are all correlational and lack direct biomechanical support. To address this research gap, we constructed, for the first time, cervical spine-implant system models after ACDF under different muscle quality conditions. These models directly reflect the effects of varying paraspinal muscle quality on adjacent segment intervertebral disc pressure, stress of cages and screws, capsular ligament stress, and cervical ROM following ACDF.

In this study, we developed for the first time a postoperative cervical spine–implant system model simulating varying muscle mass conditions after ACDF. This model directly reflects the impact of different paraspinal muscle qualities on intradiscal pressure at adjacent segments, stress distribution in the cage and screws, capsular ligament stress, and cervical ROM after ACDF surgery.

Our results indicate that degeneration of the cervical paraspinal muscles may lead to a stress-shielding effect, whereby the muscles fail to effectively share the load. As a result, a portion of the load originally borne by the paraspinal muscles is transferred to other mechanical structures, leading to increased stress on the adjacent segment discs, the cage, and screws, as well as the adjacent joint capsules (Li et al., 2025). This mechanism may contribute to the accelerated degeneration of adjacent segment discs. These findings are consistent with previous studies suggesting that atrophy of the paraspinal muscles can elevate stress on the cage and screws, indicating a correlation between muscle quality and implant-related complications (Ekşi et al., 2025).

The paraspinal muscles are key structures in maintaining the dynamic stability of the spine. The multifidus muscle, as an important stabilizer within the paraspinal muscle group, primarily relies on its deep fibers to provide spinal stability. Research by Cheng et al. suggests that a decline in paraspinal muscle quality has a negative impact on spinal stability, as reduced muscle mass leads to decreased muscular endurance, which directly affects the spine’s ability to maintain balance under load (Cheng et al., 2011). Furthermore, muscle atrophy can impair the capacity of these muscles to share loads with intervertebral discs and facet joints, thereby increasing abnormal stress distribution within spinal segments (Newell and Driscoll, 2021). Our results further validate and support these observations. Paraspinal muscle atrophy may trigger compensatory movements in adjacent segments, leading to an increased passive range of motion after cervical surgery, which is associated with reduced cervical stability. This indicates that impaired paraspinal muscle function is an important biomechanical factor influencing long-term cervical stability following ACDF.

Due to the presence of interbody implants, patients often experience reduced active cervical mobility after ACDF (Park et al., 2024). This limitation can further promote atrophy in the paraspinal muscles, particularly the multifidus, a process closely linked to the development of neck pain. Key evidence supports this: studies by Fernández (Fernández-de-las-Peñas et al., 2008) and Caffard (Caffard et al., 2024) et al. have demonstrated a correlation between preoperative paraspinal muscle quality and the severity of postoperative neck pain. Other research confirms that fatty infiltration in deep cervical extensor muscles (multifidus and semispinalis cervicis) is directly associated with mechanical neck pain (Li et al., 2023), while weakness and atrophy of these muscles represent significant risk factors for the onset and recurrence of pain (Abbott et al., 2024).

Based on this evidence, we propose the following hypothesis: reduced postoperative mobility leads to paraspinal muscle atrophy, which in turn causes myogenic pain. Persistent pain may lead to kinesiophobia (Higuchi et al., 2024), prompting patients to further limit neck movement. This reduction in activity exacerbates muscle atrophy, impairing rehabilitation outcomes and diminishing quality of life, ultimately forming a “Postoperative Cervical Muscle Dysfunction Cycle”. This cycle operates as a positive feedback loop, resulting in sustained pain and progressive functional impairment.

Therefore, based on the experimental results of this study and the above mechanistic analysis, we believe that increasing multifidus muscle mass—even as a passive structure—can enhance function and improve spinal stability by providing greater stiffness and more optimal load sharing. This simulates the biomechanical benefits conferred by increased muscle mass achieved. It can serve as an effective intervention to break this vicious cycle, thereby alleviating postoperative pain and functional impairments in patients. Consequently, in clinical practice, developing individualized and specialized postoperative rehabilitation exercise programs based on patients’ preoperative paraspinal muscle quality is crucial for ensuring the efficacy of ACDF and promoting functional recovery. This approach holds significant clinical value and should not be overlooked.

This study has several limitations. First, the constructed finite element model of the cervical spine is not sufficiently comprehensive, as it lacks components such as nerves, blood vessels, and other muscle tissues. A more complete model should be established in subsequent research. Second, the multifidus muscle tissue constructed in this study is a passive structure. The effects observed in the model resulted from the mechanical contribution of muscles as passive soft tissues. Thus, an active muscle force model should be incorporated into future studies. Third, this study only simulated changes in muscle quantity without including changes in material properties such as fatty infiltration, which will be supplemented in future research. Fourth, the model’s lower boundary is fixed at C7, while the multifidus muscle actually attaches to the thoracic vertebrae—this simplified constraint may partially limit the muscle’s impact on the overall force line. Fifth, The finite element model was built from CT data of a single healthy volunteer and lacked CSM-related degenerative changes (e.g., osteophytes, intervertebral space stenosis). Though this could impact absolute stress distribution values, the study’s core goal was to compare relative differences across muscle states—with the observed difference trend likely persisting in degenerative models. However, the specific magnitude of these differences warrants further study with patient-specific models. Additionally, due to technical constraints in model construction, there is a certain degree of deviation between the calculation results and the actual conditions. Future research should develop a more realistic and accurate finite element model and further verify its analytical results in clinical settings.

## 5 Conclusion

This study demonstrates that as muscle mass increases, the stress on adjacent segment discs and joint capsules gradually decreases, and the ROM at various cervical segments post-operation is correspondingly reduced, indicating that muscles play a critical role in stabilizing the cervical spine and supporting mechanical loads. Furthermore, the stress on the cage and screws decreases with increasing muscle mass. Therefore, we suggest that preoperative paraspinal muscle quality may have predictive value for postoperative outcomes following ACDF. Postoperative targeted muscle rehabilitation training is a key strategy for reducing biomechanical compensatory loads and is essential for maintaining long-term surgical efficacy.

## Data Availability

The original contributions presented in the study are included in the article/supplementary material, further inquiries can be directed to the corresponding authors.
